# Structure of human apurinic/apyrimidinic endonuclease 1 with the essential Mg^2+^ cofactor

**DOI:** 10.1107/S0907444913027042

**Published:** 2013-11-19

**Authors:** Brittney A. Manvilla, Edwin Pozharski, Eric A. Toth, Alexander C. Drohat

**Affiliations:** a Department of Biochemistry and Molecular Biology, University of Maryland School of Medicine, 108 North Greene Street, Baltimore, MD 21201, USA; b Pharmaceutical Sciences, University of Maryland School of Pharmacy, Baltimore, MD 21201, USA

**Keywords:** apurinic/apyrimidinic DNA, base-excision repair, nucleases, phosphoryl transfer

## Abstract

Apurinic/apyrimidinic endonuclease 1 (APE1) mediates the repair of abasic sites and other DNA lesions and is essential for base-excision repair and strand-break repair pathways. APE1 hydrolyzes the phosphodiester bond at abasic sites, producing 5′-deoxyribose phosphate and the 3′-OH primer needed for repair synthesis. It also has additional repair activities, including the removal of 3′-blocking groups. APE1 is a powerful enzyme that absolutely requires Mg^2+^, but the stoichiometry and catalytic function of the divalent cation remain unresolved for APE1 and for other enzymes in the DNase I superfamily. Previously reported structures of DNA-free APE1 contained either Sm^3+^ or Pb^2+^ in the active site. However, these are poor surrogates for Mg^2+^ because Sm^3+^ is not a cofactor and Pb^2+^ inhibits APE1, and their coordination geometry is expected to differ from that of Mg^2+^. A crystal structure of human APE1 was solved at 1.92 Å resolution with a single Mg^2+^ ion in the active site. The structure reveals ideal octahedral coordination of Mg^2+^ *via* two carboxylate groups and four water molecules. One residue that coordinates Mg^2+^ directly and two that bind inner-sphere water molecules are strictly conserved in the DNase I superfamily. This structure, together with a recent structure of the enzyme–product complex, inform on the stoichiometry and the role of Mg^2+^ in APE1-catalyzed reactions.

## Abstract 2

Human AP endonuclease 1 (APE1) belongs to the DNase I-like superfamily of enzymes that require divalent cation(s) to catalyze phosphoryl-transfer reactions. A new 1.92 Å resolution crystal structure of APE1 reveals ideal octahedral coordination of a single Mg^2+^ ion and informs on the role of this essential cofactor.

## 1. Introduction

Mammalian apurinic/apyrimidinic (AP) endonuclease 1 (APE1) is required for the repair of abasic sites and other DNA lesions and is an essential element of the base-excision repair (BER) and strand-break repair pathways (Kane & Linn, 1981 ▶; Robson & Hickson, 1991 ▶; Demple *et al.*, 1991 ▶). APE1 also has important functions in transcriptional regulation and is sometimes referred to as Ref-1 (Bhakat *et al.*, 2009 ▶). Abasic sites are mutagenic and cytotoxic lesions that arise by spontaneous rupture of the N-glycosylic bond or the action of DNA glycosylases, which excise damaged bases from DNA (Loeb, 1985 ▶; Lindahl, 1993 ▶). APE1 hydrolyzes the phosphodiester bond at abasic sites, producing 5′-deoxyribose phosphate (dRP) and the 3′-OH primer needed for repair synthesis, and is the major AP endonuclease in mammalian cells (Demple & Harrison, 1994 ▶; Robson & Hickson, 1991 ▶; Chen *et al.*, 1991 ▶). APE1 also mediates the repair of single-strand and double-strand breaks by removing 3′-blocking groups (fragmented sugar moieties), again giving a 3′-OH for subsequent repair (Izumi *et al.*, 2000 ▶; Parsons *et al.*, 2004 ▶). The repair activity of APE1 is essential for embryonic development and cell viability (Xanthoudakis *et al.*, 1996 ▶; Fung & Demple, 2005 ▶; Izumi *et al.*, 2005 ▶), and APE1 repairs damage caused by many clinically relevant anticancer agents, including AP sites and 3′-blocking groups generated by ionizing radiation and bleomycin (Chaudhry *et al.*, 1999 ▶; Parsons *et al.*, 2004 ▶; Fung & Demple, 2010 ▶; Chen & Stubbe, 2005 ▶). Thus, APE1 is an important target for the development of novel anticancer agents or adjuvants to currently used agents (Adhikari *et al.*, 2008 ▶; Abbotts & Madhusudan, 2010 ▶) and it is important to understand its structure and catalytic mechanism at the highest level of detail.

APE1 possesses a remarkable degree of catalytic power and Mg^2+^ is an essential cofactor. While Mn^2+^ can also serve as a cofactor for APE1, it is not as effective as Mg^2+^ (Kane & Linn, 1981 ▶; Barzilay *et al.*, 1995 ▶). The AP endonuclease activity of APE1 is potent, with a maximal rate for the chemical step of at least 850 s^−1^ (Maher & Bloom, 2007 ▶). In contrast, the non-enzymatic hydrolysis of phosphodiester bonds in DNA is extremely slow, occurring with a half-life of 100 000 years (Radzicka & Wolfenden, 1995 ▶). Thus, APE1 exhibits a rate enhancement of 10^16^ for its endonuclease activity. Previous studies show that this robust activity can be fully suppressed by rigorous chelation of Mg^2+^ (Erzberger & Wilson, 1999 ▶; Maher & Bloom, 2007 ▶), indicating that this cofactor is essential for catalysis. Notably, APE1 belongs to the large DNase I superfamily of endonuclease, exonuclease and phosphatase (EEP) enzymes (Dlakić, 2000 ▶). The important catalytic residues are strictly conserved in this superfamily, including several that coordinate Mg^2+^ (or other divalent cation) either directly or by an inner-sphere water molecule. However, the stoichiometry and catalytic function of the divalent cation remain unresolved for APE1 and other enzymes in the DNase I superfamily. Identifying the binding sites and function(s) of metals remains an important problem in biology (Yannone *et al.*, 2012 ▶).

To date, three different crystal structures have been reported in the literature for DNA-free human APE1, but they contain a surrogate metal rather than the native Mg^2+^ cofactor. A structure of Mg^2+^-bound APE1 was deposited in the Protein Data Bank in 2011 (PDB entry 3u8u; R. Agarwal & M. D. Naidu, unpublished work), but no paper describing this structure has appeared in the literature. The first reported structure contained one Sm^3+^ ion in the active site (Gorman *et al.*, 1997 ▶) and this was taken to represent the binding site for Mg^2+^, based on similarity to a structure of Mn^2+^-bound exonuclease III, the prokaryotic homolog of APE1 (Mol *et al.*, 1995 ▶). The metal-binding site identified in this first APE1 structure, often referred to as the ‘A’ site, is consistent with the binding site observed for Mg^2+^ or other divalent metals in structures of other enzymes within the large DNase I superfamily (Suck & Oefner, 1986 ▶; Dlakić, 2000 ▶). Subsequently, two additional structures of DNA-free APE1 were reported with Pb^2+^ ions in the active site (Beernink *et al.*, 2001 ▶). Both of these structures have a Pb^2+^ ion in the A site and one has a second Pb^2+^ ion, in a location that is referred to as the ‘B’ site, coordinated by three residues that are essential for catalysis and strictly conserved in the DNase I superfamily (Asp210, Asn212 and His309; Beernink *et al.*, 2001 ▶). This finding raised the possibility that APE1 and related enzymes require two divalent metals for catalysis. In this two-metal mechanism, the B-site metal would serve to stabilize the hydroxide nucleophile, *i.e.* lower the p*K*_a_ for the nucleophilic water molecule. However, subsequent studies found that Pb^2+^ is a potent inhibitor of APE1 (McNeill *et al.*, 2004 ▶, 2007 ▶) and this inhibition could reflect a perturbation of the catalytic function of one or more of these three essential residues. Moreover, the ligands and coordination geometry are expected to differ for Sm^2+^ and Pb^2+^ compared with Mg^2+^.

Accordingly, a structure of DNA-free APE1 with the preferred Mg^2+^ ion is needed to understand the catalytic role of this essential cofactor for APE1 and related DNase I-like enzymes. This notion is underscored by continuing controversy as to whether the DNase I superfamily enzymes require one or two Mg^2+^ (or Ca^2+^) ions for catalysis (Gao *et al.*, 2012 ▶; Tsutakawa *et al.*, 2013 ▶; Jones *et al.*, 1996 ▶; Parsiegla *et al.*, 2012 ▶) and the proposal that a single Mg^2+^ cofactor moves from the A site in the free enzyme to the B site in the enzyme–substrate (ES) complex during catalysis (Oezguen *et al.*, 2007 ▶, 2011 ▶). Here, we report the crystal structure of human APE1 solved at 1.92 Å resolution with a single Mg^2+^ ion bound in the active site. This new structure, together with the recently reported structure of an enzyme–product (EP) complex of APE1, which also contains a single active-site Mg^2+^ (Tsutakawa *et al.*, 2013 ▶), informs on the mechanism of this critical repair enzyme.

## 2. Experimental procedures

### 2.1. Protein preparation and crystallization

Human APE1 lacking the 38 N-terminal residues, APE1^ΔN38^, was expressed in *Escherichia coli* and purified (at 4°C) as described previously (Manvilla *et al.*, 2009 ▶). The sample used for crystallization consisted of APE1^ΔN38^ (∼9 mg ml^−1^) in a neutral-pH buffer consisting of 0.01 *M* HEPES pH 7.5, 0.025 *M* NaCl, 1 m*M* DTT, 1 m*M* MgCl_2_. Crystals were grown by vapor diffusion at 22°C in sitting-drop format using 0.5 µl protein sample and 1.5 µl mother liquor, which consisted of 0.1 *M* MES pH 6.5, 30%(*v*/*v*) PEG 300. Crystals were grown for 3–5 d, the mother liquor was supplemented with 20% glycerol and the crystals were flash-cooled in liquid nitrogen.

### 2.2. Data collection, structure determination and refinement

X-ray diffraction data were collected on beamline 7-1 of the Stanford Synchrotron Radiation Lightsource (SSRL). The images were processed and scaled using *MOSFLM* (Leslie & Powell, 2007 ▶) and *AIMLESS* (Evans, 2011 ▶) from the *CCP*4 program suite (Winn *et al.*, 2011 ▶). Initial phases were obtained by molecular replacement using the program *Phaser* (McCoy *et al.*, 2005 ▶) with a previously determined crystal structure of APE1 (PDB entry 1bix; Gorman *et al.*, 1997 ▶) as the search model. Model building was performed with *Coot* (Emsley & Cowtan, 2004 ▶) and model refinement was performed using *REFMAC* (Winn *et al.*, 2001 ▶). The final coordinates were deposited in the Protein Data Bank as entry 4lnd. Structural figures were prepared with *PyMOL* (DeLano, 2002 ▶).

## 3. Results and discussion

### 3.1. Overall structure of the APE1 holoenzyme

A construct of human APE1 that lacks the 38 N-terminal residues, APE1^ΔN38^ (Manvilla *et al.*, 2009 ▶), was crystallized under neutral pH conditions by sitting-drop vapor diffusion (at 22°C). The crystals belonged to the monoclinic space group *C*2, with three molecules per asymmetric unit (Table 1 ▶). The structure was solved by molecular replacement and refined to a crystallographic *R* factor of 21.7% and an *R*_free_ of 24.1% at a resolution of 1.92 Å (Table 1 ▶), and includes all but the 43 N-­terminal residues of human APE1. We note that the 40–43 N-­terminal residues have been absent in all crystal structures determined to date for APE1, either free or DNA-bound, even when the full-length enzyme was used for crystallization (Beernink *et al.*, 2001 ▶). This is consistent with our findings from NMR studies that these residues are disordered in solution (Manvilla *et al.*, 2009 ▶, 2011 ▶). Previous studies indicated that the 40 N-terminal residues are dispensable for the repair and the redox activities of APE1 (Izumi & Mitra, 1998 ▶).

The structure presented here is similar in overall fold to the previously reported structures of DNA-free APE1, as expected, but there are several important differences, as summarized in Table 2 ▶. Ours is the first reported structure of the DNA-free enzyme with the essential Mg^2+^ cofactor bound in the active site, and it reveals the detailed mechanism and geometry for Mg^2+^ coordination, as discussed below. While some residues within the structured catalytic domain are absent in previously reported structures of DNA-free APE1, electron density is observed for all residues from Leu44 to the C-terminus (Leu318) in the structure reported here. When compared with the previously determined Sm^3+^-bound and Pb^2+^-bound structures, the new structure here is most similar to the structure containing a single Pb^2+^ ion in the active site and least similar to the structure with two Pb^2+^ ions (Table 2 ▶). While the single-Pb^2+^ structure is of relatively high resolution (1.95 Å; Beernink *et al.*, 2001 ▶), the crystals were grown under acidic conditions (pH 4.6) and the structure lacks 17 residues in the catalytic domain of the enzyme (Table 2 ▶). For these reasons, and others detailed below, the structure reported here provides a substantial advance over the previously reported structures of DNA-free APE1.

### 3.2. Octahedral coordination of the single Mg^2+^ ion

**Figure 1:**
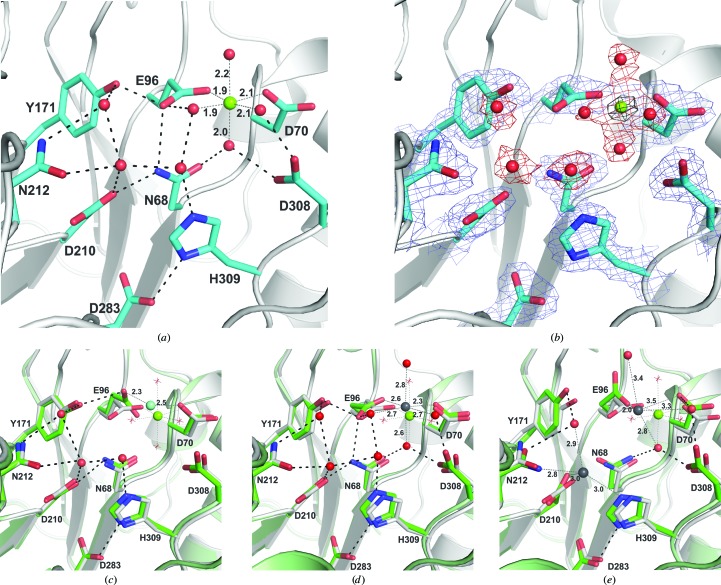
New crystal structure of human APE1 with the native Mg^2+^ cofactor and previous structures with surrogate metals. (*a*) Close-up view of the active site of the new structure, showing Mg^2+^ and several ordered water molecules (molecule *A* of the three molecules in the asymmetric unit for PDB entry 4lnd). Also shown are the important catalytic residues; all but Asp70 are strictly conserved in the DNase I superfamily (Tyr171 is replaced by His in some members). The octahedral coordination of Mg^2+^ is indicated by dotted lines with distances provided (also given in Table 3 ▶). Hydrogen bonds are indicated by dashed lines. (*b*) The same view of the active site showing a 2*F*_o_ − *F*_c_ OMIT map contoured at 1.5σ for protein and waters and an *F*_o_ − *F*_c_ OMIT map contoured at 8.0σ for the Mg^2+^ ion (black mesh). Note that one or more of the three non-Mg^2+^-coordinating water molecules are not observed in the other two protein molecules (*B* and *C*) in the asymmetric unit. (*c*) Previously reported structure of Sm^3+^-bound APE1 (green; PDB entry 1bix; Gorman *et al.*, 1997 ▶) aligned with the new Mg^2+^-bound structure (white). The coordination of the Sm^3+^ ion (cyan) is shown (dotted lines) with distances. The coordination of Mg^2+^ (green) in the new structure is also indicated (without distances). Water molecules shown as red spheres and hydrogen bonds (dashed lines) are for the Sm^3+^-bound structure. The water molecules that coordinate Mg^2+^ are shown as red stars. (*d*) The previously reported structure of APE1 with one Pb^2+^ ion (green; PDB entry 1hd7; Beernink *et al.*, 2001 ▶) aligned with the new Mg^2+^-bound structure (white). The coordination of the Pb^2+^ ion (gray) is shown (dotted lines) with distances and the coordination of Mg^2+^ (green) is also indicated. Water molecules (red spheres) and hydrogen bonds (dashed lines) are for the Pb^2+^-bound structure (waters that coordinate Mg^2+^ are shown as red stars). (*e*) The previously reported structure of APE1 with two Pb^2+^ ions (green; PDB entry 1e9n; Beernink *et al.*, 2001 ▶) aligned with the new Mg^2+^-bound structure (white). The coordination of the Pb^2+^ ions (gray) is shown (dotted lines) with distances and the coordination of Mg^2+^ (green) is similarly indicated. Water molecules (red spheres) and hydrogen bonds (dashed lines) are for the Pb^2+^-bound structure (waters that coordinate Mg^2+^ are shown as red stars).

As shown in Fig. 1 ▶(*a*), the new APE1 structure reveals a single Mg^2+^ ion in the active site, coordinated in ideal octahedral geometry by two carboxylate groups (Asp70 and Glu96) and four water molecules. Fig. 1 ▶(*a*) also shows six additional active-site groups that are strictly conserved in the DNase I superfamily (Tyr171 is replaced by His in some cases) and three additional water molecules that are bound by these residues. Fig. 1 ▶(*b*) shows the same view with an *F*_o_ − *F*_c_ OMIT map contoured at 8.0σ for the Mg^2+^ ion and a 2*F*_o_ − *F*_c_ OMIT map contoured at 1.5σ for the side chains and water molecules. The same octahedral metal-coordination geometry and ligands are observed in all three active sites of the asymmetric unit. The Mg^2+^-coordination distances for each of the three sites in the asymmetric unit are provided in Table 3 ▶; they are in good agreement with the Mg^2+^-coordination distances in high-­resolution (≤1.25 Å) protein crystal structures and compounds in the Cambridge Structural Database (2.07 ± 0.05 Å for water as a ligand, 2.07 ± 0.10 Å for a carboxylate ligand; Harding, 2006 ▶). The binding site for Mg^2+^ in the new structure is the same as that observed for the surrogate metals Sm^3+^ and Pb^2+^ in previous structures of DNA-free APE1 (Figs. 1 ▶ *c*, 1 ▶ *d* and 1 ▶ *e*) and this site is typically referred to as the ‘A site’. However, important differences are observed regarding the mechanism and geometry of metal coordination, as discussed below.

We find no evidence for a second Mg^2+^ ion at the ‘B site’, which was observed to bind a second Pb^2+^ ion *via* direct coordination through the side chains of Asp210, Asn212 and His309 in a previous APE1 structure (Fig. 1 ▶ *e*; Beernink *et al.*, 2001 ▶). Rather, a water molecule occupies this site in the structure described here, forming hydrogen bonds to the side chains of Asp210 and Asn212 and two other active-site water molecules (Fig. 1 ▶ *a*); the contact distances (≥2.6 Å) and geometry indicate that it is not a second Mg^2+^ ion. The observation that DNA-free APE1 binds a single Mg^2+^ ion at the A site (Asp70 and Glu96) and that a second Mg^2+^ ion does not bind at the B site is consistent with the results of two previous NMR studies (Lowry *et al.*, 2003 ▶; Lipton *et al.*, 2008 ▶). While it is possible that a second Mg^2+^ ion could enter the B site with the DNA substrate, this seems unlikely given that a second Mg^2+^ ion is not observed in either of the two existing structures of the APE1 enzyme–product complex (Mol *et al.*, 2000 ▶; Tsutakawa *et al.*, 2013 ▶).

**Figure 2:**
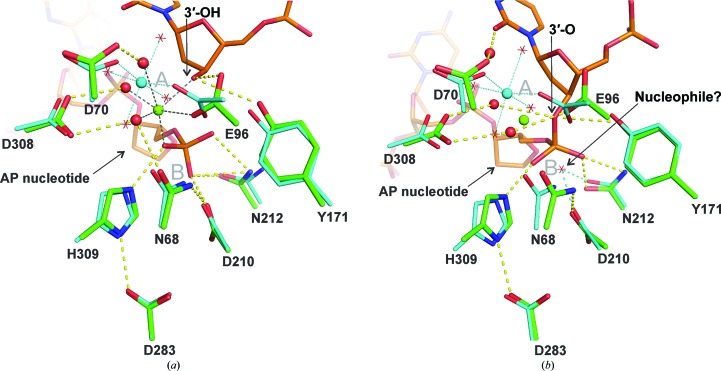
Alignment of the new Mg^2+^-bound APE1 structure with DNA-bound structures. (*a*) Recently determined structure of the APE1 enzyme–product (EP) complex (green; PDB entry 4iem; Tsutakawa *et al.*, 2013 ▶) aligned with the structure of Mg^2+^-bound APE1 (cyan) reported here. DNA from the EP complex (orange) contains a 3′-OH and a 5′-deoxyribose phosphate (dRP). The Mg^2+^ ion from the EP complex is colored green and its coordination is indicated by black dotted lines. The Mg^2+^ ion from the new DNA-free structure is colored cyan and its coordination is indicated by cyan dotted lines, with coordinating water molecules shown as red stars. Hydrogen-bond interactions (yellow dashes) are shown for the EP complex only. The approximate locations of the A and B sites are noted (gray symbols). (*b*) Structure of the APE1 enzyme–substrate (ES) complex (green; PDB entry 1dew; Mol *et al.*, 2000 ▶) aligned with the new structure of Mg^2+^-bound APE1 (cyan). The DNA in the ES complex (orange) contains an intact abasic site and Mg^2+^ was omitted from the ES complex to halt P—O bond cleavage. The green sphere indicates the position of the Mg^2+^ ion in the EP complex (also aligned with DNA-free APE1). Mg^2+^ in the DNA-free structure is shown in cyan; its coordination is indicated by cyan dotted lines, with coordinating waters shown as red stars. Hydrogen-bond interactions (yellow dashes) are shown for the ES complex. The potential nucleophilic water from our DNA-free structure is shown as a red star, with cyan dashes indicating hydrogen bonds to Asp210 and Asn212. This water molecule was also observed in previous structures of APE1 with Sm^3+^ or a single Pb^2+^ ion (Fig. 1 ▶). The approximate locations of the A and B sites are noted (gray symbols).

One of the two residues that directly coordinate the Mg^2+^ ion, Glu96, is strictly conserved in the DNase I superfamily, while the other, Asp70, seems to be restricted to mammalian APE1 (Gorman *et al.*, 1997 ▶; Castillo-Acosta *et al.*, 2009 ▶). We note that Glu96 also directly coordinates the single Mg^2+^ ion that was observed in a recently reported enzyme–product (EP) complex of APE1, while the Asp70 carboxylate binds a water molecule that in turn coordinates Mg^2+^ (Fig. 2 ▶ *a*; Tsutakawa *et al.*, 2013 ▶). Previous studies found that the E96Q mutation causes a 2300-fold loss in AP endonuclease activity (Erzberger & Wilson, 1999 ▶), consistent with an important role of Glu96 in coordinating the Mg^2+^ cofactor. An important role of Asp70 is indicated by the 26-fold loss in endonuclease activity of the D70A or D70R mutations (Erzberger & Wilson, 1999 ▶). Moreover, D70A and E96Q variants exhibit diminished endonuclease activity relative to native APE1 under conditions of limiting Mg^2+^ (Nguyen *et al.*, 2000 ▶; Castillo-Acosta *et al.*, 2009 ▶).

In addition to the two carboxylate groups, the Mg^2+^ cofactor is coordinated by four water molecules, three of which are bound by the side chains of Asn68, Glu96 and Asp308 (Fig. 1 ▶ *a*). All of these residues are strictly conserved in the DNase I superfamily and, as noted above, Glu96 also coordinates Mg^2+^ directly. The catalytic importance of Asn68 is indicated by the 600-fold loss in AP endonuclease activity caused by the N68A mutation (Nguyen *et al.*, 2000 ▶). In addition to coordinating Mg^2+^ (indirectly) in the free enzyme and in the EP complex (Fig. 2 ▶ *a*), Asn68 contacts the essential carboxylate group of Asp210 and could potentially facilitate its role in catalysis (Fig. 1 ▶ *a*). The carboxylate of Asp308 binds two of the four inner-sphere water molecules of Mg^2+^. While the D308A variant exhibits a relatively modest fivefold loss in AP endonuclease activity, it has a much greater sensitivity to low Mg^2+^ conditions than does native APE1 (Erzberger & Wilson, 1999 ▶; Nguyen *et al.*, 2000 ▶).

The coordination mechanism for Mg^2+^ observed in the new structure differs substantially from that observed for Sm^3+^ and Pb^2+^ in previous structures of DNA-free APE1. While the APE1 structure with Sm^3+^ suggested that Asp70 and Glu96 could directly coordinate the Mg^2+^ ion (Gorman *et al.*, 1997 ▶), the four water molecules that coordinate Mg^2+^ in the new structure are not found in the Sm^3+^-bound structure (Fig. 1 ▶ *c*). Another structure, determined under acidic conditions (pH 4.6), contains a single Pb^2+^ ion that exhibits octahedral coordination involving Asp70, Glu96 and four water molecules (Fig. 1 ▶ *d*; Beernink *et al.*, 2001 ▶), similar to the coordination of Mg^2+^ found in the structure reported here. However, the metal–oxygen distances are substantially longer for Pb^2+^ relative to Mg^2+^ (Figs. 1 ▶ *a* and 1 ▶ *d*). A third structure, determined at pH 7.5, contained two Pb^2+^ ions, one at the A site and the other at the B site, coordinated by Asp210, Asn212 and His309 (Fig. 1 ▶ *e*). As such, the second Pb^2+^ ion displaces the water molecule that is bound by Asp210 and Asn212 in all of the single-metal structures of DNA-free APE1 (Figs. 1 ▶ *a*, 1 ▶ *c* and 1 ▶ *d*). As discussed below, this water molecule seems to be a good candidate for the nucleophile in the hydrolytic reaction catalyzed by APE1. Again, we observe no evidence for Mg^2+^ at the B site in the structure reported here, consistent with findings that imidazole side chains are not a common ligand for Mg^2+^ ions (Harding, 2001 ▶, 2006 ▶). The coordination geometry for Pb^2+^ in the A site of the two-Pb^2+^ structure (Fig. 1 ▶ *e*) is non-ideal when compared with the octahedral geometry observed in structures with a single Pb^2+^ ion (Fig. 1 ▶ *d*) or a single Mg^2+^ ion (Fig. 1 ▶ *a*). Thus, our structure, together with previous structures, suggests that Pb^2+^ inhibits APE1 by binding to this B site and perturbing the function of one or more of the three essential catalytic residues (Asp210, Asn212 or His309) and/or by perturbing binding of Mg^2+^ at the A site.

### 3.3. Coordination of Mg^2+^ in the free enzyme and the enzyme–product complex

The structure of Mg^2+^-bound APE1 reported here, together with the recently determined structure of an enzyme–product (EP) complex (2.4 Å resolution; Tsutakawa *et al.*, 2013 ▶), allows a detailed comparison of the position and coordination mechanism for the essential Mg^2+^ cofactor at two steps along the catalytic pathway. Shown in Fig. 2 ▶(*a*) is an alignment of these structures, with active-site residues colored cyan for DNA-free APE1 and green for the EP complex and with DNA colored orange. For the EP complex, the enzyme active site contains a single Mg^2+^ ion (green sphere) and DNA with a hydrolyzed phosphodi­ester bond, *i.e.* 3′-OH and 5′-deoxy­ribose phosphate (dRP). The conserved catalytic residues are positioned nearly identically in the aligned structures, except for Glu96, which repositions between the free enzyme and EP complex, predominantly *via* rotation about χ_1_, with little change in the main chain. Notably, the conformation of Glu96 observed in the EP (and ES) complex is similar to that observed in the structure of DNA-free APE1 with two Pb^2+^ ions (Fig. 1 ▶ *e*) and in some of the six molecules in the asymmetric unit of the unpublished structure of Mg^2+^-bound APE1 (not shown). Although the orientation of Glu96 is the same for all three active sites in the asymmetric unit for our Mg^2+^-bound structure (Fig. 1 ▶ *a*), these observations suggest the possibility of conformational sampling for Glu96 in the DNA-free enzyme. The Mg^2+^ ion is positioned relatively similarly in the DNA-free enzyme (cyan sphere) and the EP complex (green sphere), with a separation of 2.2 Å, suggesting that the cofactor could exhibit minimal movement during catalysis. As discussed above, we find that DNA-free APE1 coordinates Mg^2+^ *via* the carboxylate groups of Asp70 and Glu96 and four water molecules. In the EP complex, Mg^2+^ is coordinated directly by Glu96 (carboxylate), the 3′-OH (leaving group), a nonbridging O atom of the nascent 5′-phosphate and by three water molecules, one of which is bound to Asp70 (Tsutakawa *et al.*, 2013 ▶). Thus, one carboxylate O atom of Glu96 contacts Mg^2+^ in both the free enzyme and the EP complex, while the other carboxylate O atom of Glu96 trades a contact with water in the free enzyme for a contact with the 3′-OH in the EP complex (Fig. 2 ▶ *a*). These observations suggest that Mg^2+^ might not need to move far from its location in the free enzyme to perform its catalytically essential function(s), which could include positioning of the target phosphate, polarization of the scissile P—O bond and stabilization of the negative charge developing at one of the nonbridging O atoms and/or the 3′-­oxygen in the transition state (Gorman *et al.*, 1997 ▶; Mol *et al.*, 2000 ▶; Herschlag *et al.*, 1991 ▶).

### 3.4. Alignment of the Mg^2+^-bound enzyme with the enzyme–substrate complex

It is also informative to compare the new structure of Mg^2+^-bound APE1 with a previously reported structure of the enzyme–substrate (ES) complex, which was crystallized in the absence of Mg^2+^ in order to preclude hydrolysis of the phosphodiester bond (Mol *et al.*, 2000 ▶). Shown in Fig. 2 ▶(*b*) is an alignment of these structures, with active-site residues colored cyan for the free enzyme and green for the ES complex and with DNA colored orange. In the ES complex, the DNA backbone is intact and the abasic nucleotide is flipped into the active site. For reference, the Mg^2+^ ion from the EP complex (Tsutakawa *et al.*, 2013 ▶) is shown as a green sphere and seems to be well positioned with respect to the substrate DNA to perform one or more of the catalytic functions mentioned above. Previous solid-state ^25^Mg NMR studies found two distinct environments for a single Mg^2+^ ion in the ES complex, suggesting that the cofactor is disordered (Lipton *et al.*, 2008 ▶). This could reflect conformational exchange of the Mg^2+^ ion in the ES complex, perhaps between its location in the free enzyme and a site similar to that observed in the EP complex (Fig. 2 ▶ *b*).

Although no candidate nucleophile is observed in the crystal structure of the ES complex (Mol *et al.*, 2000 ▶), a water molecule coordinated by Asp210 and Asn212 in the DNA-free structure reported here seems to be well positioned to serve as the nucleophile for hydrolysis of the phosphodiester bond (Figs. 1 ▶ *a* and 2 ▶ *b*). The idea that the nucleophile may be bound by Asp210 and Asn212 and activated by Asp210 is supported by recent molecular-dynamics studies (Tsutakawa *et al.*, 2013 ▶). The Asp210/Asn212-bound water molecule was observed in previously determined structures of DNA-free APE1, except the structure with two Pb^2+^ ions, where the potential nucleophile is displaced by the second Pb^2+^ ion (Figs. 1 ▶ *c*, 1 ▶ *d* and 1 ▶ *e*; Beernink *et al.*, 2001 ▶). As noted, binding of Pb^2+^ to the B site could account for the finding that Pb^2+^ is a potent inhibitor of APE1. Our structure also indicates that a water molecule binds to the imidazole of His309 (in one of the three active sites in the asymmetric unit), and the same water is observed in the DNA-free structures with Sm^3+^ or a single Pb^2+^ ion (Fig. 1 ▶) and in the unpublished Mg^2+^ structure (in four of the six molecules in the asymmetric unit; not shown). This is notable because it has been proposed that His309 acts as a general base to activate the water nucleophile in reactions catalyzed by APE1 and related enzymes (Gorman *et al.*, 1997 ▶; Mol *et al.*, 1995 ▶; Lipton *et al.*, 2008 ▶; Suck & Oefner, 1986 ▶). However, the His309-bound water seems to be poorly positioned for nucleophilic attack in the aligned structures (free E and ES complex) when compared with the Asp210-bound water (Fig. 2 ▶ *b*), and His309 seems to be poorly positioned to act as the essential general base. For this and other reasons, we favor the hypothesis that Asp210 activates the nucleophile (Tsutakawa *et al.*, 2013 ▶; Mol *et al.*, 2000 ▶). Nevertheless, the observation that the Asp210-bound and His309-bound waters are not seen in all three active sites of the asymmetric unit in the new structure suggests that this network of active-site water molecules is dynamic and that the position of these water molecules in the DNA-free enzyme will likely change upon formation of the ES complex. Additional studies are needed to establish the mechanism of nucleophile activation for APE1 and other members of the DNase I superfamily.

The crystal structure with two Pb^2+^ ions raised the possibility that a second Mg^2+^ ion binds to the B site (Asp210, Asn212 and His309) in the ES complex, where it might stabilize a hydroxide ion for nucleophilic attack (Beernink *et al.*, 2001 ▶). However, removal of the D210A and Asn212 side chains by mutagenesis leads to huge (10^4^–10^5^-fold) losses in steady-state AP endonuclease activity (*i.e.* *k*_cat_; Erzberger & Wilson, 1999 ▶; Rothwell & Hickson, 1996 ▶; Rothwell *et al.*, 2000 ▶). As noted previously, the mutational effect on *k*_cat_ is likely to underestimate the effect on the chemical step; *k*_cat_ is strongly limited by a step after the chemistry for wild-type APE1, while it is likely to report on the chemical step for severely impaired mutants (Maher & Bloom, 2007 ▶). Indeed, single-turnover experiments suggest that the D210A mutation slows the chemical step by up to 10^8^-fold (Maher & Bloom, 2007 ▶). The observation of such severe damaging effects seems more consistent with a direct role for Asp210 and Asn212 in binding and activating the nucleophile rather than coordinating a catalytic Mg^2+^ ion. As noted above, our structure (1.9 Å resolution) and previously determined structures of free APE1 (except for the two-Pb^2+^ structure) contain a water molecule at the ‘B’ site of the DNA-free enzyme and a second Mg^2+^ is not observed in the recently determined structure of the EP complex (2.4 Å resolution). Together, these observations argue against the idea that Mg^2+^ binds to both the A and B sites or that a single Mg^2+^ moves from the A site in the free enzyme to the B site in the ES complex to activate the water nucleophile and then back to the A site in the EP complex (Oezguen *et al.*, 2007 ▶, 2011 ▶).

## 4. Concluding remarks

The structure presented here is of the highest resolution reported for human APE1 to date, was solved at neutral pH, includes all residues in the catalytic domain and reveals the detailed mechanism for octahedral coordination of the essential Mg^2+^ cofactor. The results provide new insight into the role of divalent metal-ion cofactors in the large and diverse DNase I superfamily of enzymes that catalyze phosphoryl-transfer reactions. This new structure, and the approach employed to obtain high-resolution structures with the native Mg^2+^ cofactor, could be useful for the design and optimization of inhibitors of APE1, an essential repair enzyme that is an important target for novel anticancer agents and adjuvants to existing agents (Adhikari *et al.*, 2008 ▶; Abbotts & Madhusudan, 2010 ▶).

## Supplementary Material

PDB reference: APE1, 4lnd

## Figures and Tables

**Table 1:** Data-collection and refinement statistics for APE1^ΔN38^ Values in parentheses are for the highest resolution shell.

Data collection	
Space group	*C*2
Unit-cell parameters (Å, °)	*a* = 165.8, *b* = 90.9, *c* = 94.0, α = 90.0, β = 123.2, γ = 90.0
Resolution (Å)	48.0–1.92 (1.96–1.92)
*R*_p.i.m._	0.069 (0.482)
Mean *I*/σ(*I*)	3.6 (0.7)
CC_1/2_	0.991 (0.745)
Completeness (%)	85.4 (84.4)
Multiplicity	2.5 (2.4)
Wilson *B* factor (Å^2^)	25.1
Refinement	
Resolution (Å)	48.0–1.92
No. of reflections	71263
*R*_work_/*R*_free_ (%)	21.7/24.1
No. of atoms	
Total	6443
Protein	6320
Waters	120
Metal ions	3
*B* factors (Å^2^)	
Protein	34.7
Waters	32.5
Metal ions	35.0
Ramachandran plot† (%)	
Favored regions	90.1
Allowed regions	9.8
Generously allowed regions	0.1
Outliers	0.0
R.m.s.d.	
Bond lengths (Å)	0.008
Bond angles (°)	1.243

† The Ramachandran analysis was performed using *PROCHECK* (Laskowski *et al.*, 1993 ▶).

**Table 2:** Comparison of data-collection and refinement statistics for APE1 structures

PDB entry	1bix	1hd7	1e9n	3u8u †	4lnd
pH of crystallization	6.2	4.6	7.5	7.5	6.5
Resolution (Å)	2.20	1.95	2.20	2.15	1.92
Space group	*C*2	*C*2	*C*2	*P*2_1_	*C*2
Molecules per asymmetric unit	1	1	2	6	3
Residues absent from structure‡	100–104	102–112, 122–127	124–125	None§	None
Metal(s) in active site	Sm^3+^	Pb^2+^	Pb^2+^ (2)	Mg^2+^	Mg^2+^
R.m.s.d. to new structure¶ (Å)					
Molecule *A*	0.293	0.280	0.363	0.219–0.309	N/A
Molecule *B*	0.321	0.307	0.383	0.225–0.315	N/A
Molecule *C*	0.271	0.265	0.353	0.220–0.284	N/A

† The structure was deposited in the Protein Data Bank (PDB entry 3u8u) but has not been reported in the literature.

‡ All structures of APE1 lack the N-terminal residues up to residue ∼40.

§ Some residues (123–128) are missing in two of the six molecules in the asymmetric unit.

¶ R.m.s.d. determined from pairwise alignment (all atoms) using *PyMOL* (DeLano, 2002 ▶). Values are given for each of the three molecules in the asymmetric unit for the new structure reported here (PDB entry 4lnd). For PDB entry 1e9n the values shown for molecule *A* are nearly identical (±0.5%) to those for molecule *B*. For PDB entry 3u8u the range of r.m.s.d. values is given for aligning each of the three molecules in the asymmetric unit of our structure (PDB entry 4lnd) with each of the six protein molecules in the asymmetric unit of PDB entry 3u8u.

**Table 3:** Mg^2+^-coordination distances (Å) for each molecule of the asymmetric unit Interatomic distances (Å) between the Mg^2+^ ion and the six coordinating ligands (Fig. 1 ▶ *a*). Three of the four coordinating water molecules are denoted by the residue(s) to which they are bound (in parentheses). The ‘upper’ H_2_O refers to the uppermost H_2_O ligand in Fig. 1 ▶.

	Molecule		
Ligand	*A*	*B*	*C*
H_2_O (upper)	2.20	2.08	2.14
H_2_O (Glu96)	1.94	2.07	2.03
H_2_O (308)	2.07	2.10	2.12
Asp70	2.14	2.04	1.94
Glu96	1.95	1.98	1.92
H_2_O (Asn68, Glu308)	2.00	2.16	2.11
